# Novel contributions in canine craniometry: Anatomic and radiographic measurements in newborn puppies

**DOI:** 10.1371/journal.pone.0196959

**Published:** 2018-05-08

**Authors:** Maria Elena Andreis, Umberto Polito, Maria Cristina Veronesi, Massimo Faustini, Mauro Di Giancamillo, Silvia C. Modina

**Affiliations:** 1 Department of Health, Animal Science and Food Safety, Università degli Studi di Milano, Milano, Italy; 2 Department of Veterinary Medicine, Università degli Studi di Milano, Milano, Italy; Faculty of Animal Sciences and Food Engineering, University of São Paulo, BRAZIL

## Abstract

The largest differences in intraspecific head shape among the *Carnivora* order are to be found in dogs. Based on their skull morphotypes, dog breeds are currently classified as dolichocephalic, mesaticephalic and brachycephalic. Due to the fact that some breeds have not been yet defined, this classification is incomplete; moreover, multi-breed studies on the skull morphology of puppies have never been performed. The aim of this work was to verify (i) whether differences in the skull conformation of purebred puppies are already present within the first week of age; (ii) whether radiographic and anatomic measures could be considered interchangeable, and (iii) to possibly classify puppies from non-categorized breeds thanks to their radiographic cranial measurements using neural nets. One hundred and thirty-seven dead puppies aged 0–7 days were examined considering their anatomic and radiographic measures. All linear measures and anatomic indices significantly differed among brachycephalic and non-brachycephalic puppies. Radiographic indices, with the exception of CI, identified the three skull morphotypes (*p*<0.05, for all comparisons). Radiographic and anatomic measures proved to be non-interchangeable in newborn puppies. Finally, nineteen puppies belonging to 5 non-categorized breeds could be classified thanks to neural nets in the three skull morphotypes with different probability (P between 0,66 and 0,95).

## Introduction

The phenotypic differences existing within the canine species can be well-represented by their skull shape. Although some heterogeneity in skull shape and size is found within the *Carnivora* order [1, 2], *Canis lupus familiaris* exhibits the largest intraspecific differences [3, 4], mainly due to human selection. In fact, particularly during the last two centuries, dogs have been selected according to the shape of their skull on account of attitudinal traits, personal taste or common trends, to exceed the significant number of 200 breeds [http://www.thekennelclub.org.uk/]. Currently, dog breeds are classified as dolichocephalic, mesaticephalic and brachycephalic based on morphological ratios that consider the neurocranium and/or the splanchnocranium [5–16]. As a general rule, dolichocephalic dogs show a greater development of the skull longitudinal axis; brachycephalic dogs have a shorter and larger skull, and mesaticephalic dogs exhibit intermediate skull features. This traditional craniometry-based classification is still used despite the outcomes of several recent studies based on genetics performed also to investigate the canine skull pattern derived from the wolf [1, 17, 18, 19, 20].

A better characterization of all the different phenotypes was gained after several morphometric and allometric studies together with reference values obtained from anatomic and radiographic linear measures and derived indices. In literature, most linear measures are anatomical and performed on the skull deprived of the soft tissues [1, 2, 12, 21–23]. Other data are based on the observation of living subjects [24, 25] or on measures from pictures [26–28]. Moreover, imaging studies on living animals by radiography [15, 29] or Computed Tomography [14] have been performed. Nevertheless, not all dog breeds have been classified unanimously on the basis of their skull morphology. In fact, while some breeds fall within defined categories, some others are still unclassified. The different techniques employed for skull measurements (anatomical, photographic or radiographic) may account for this heterogeneity and the variation of breed standards along time, depending on human selection, may as well have influenced the results. Moreover, some authors do not agree with the imposition of strict categories and propose a continuous spectrum of skull shapes ranging from extreme brachycephaly (e.g. Chihuahua) to extreme dolichocephaly (e.g. Borzoi) based on the cephalic index [26–28, 30–34].

Noticeably, most veterinarian craniometric studies have been performed on adult animals, so no detailed information is currently available on growing dogs. To the authors’ knowledge, the only exception is represented by two studies on German Shepherd puppies [22, 23]. However, it has been postulated that in brachycephalic breeds the skull shape is generated before birth and continues its development after birth [35]. Recently, an allometric study was performed on newborn puppies belonging to small-sized breeds. The study included craniometric measures, but skull morphotype was not considered [36]. Since literature lacks study design standardization, the present investigation aimed to evaluate skull morphometry in newborn dogs and to classify puppies belonging to previously non-categorized canine breeds. In particular, it was conducted to find out any possible (i) difference in craniometric measures between newborn puppies belonging to dolichocephalic, mesaticephalic and brachycephalic breeds; (ii) interchange of craniometric anatomical measures performed on newborn puppies with radiological measures, and (iii) classification as dolichocephalic, mesaticephalic or brachycephalic for newborn puppies belonging to non-categorized breeds.

## Materials and methods

### Animals

Puppies under examination were obtained from breeders signing a prior informed consent, and the research was approved by the Animal Welfare Body of the University of Milan (AWB/OPBA, 58/2016). They all aged 0–7 days and were clinically evaluated by one of the authors, a Diplomate at the European College of Animal Reproduction (MCV). They were born full term, after normal pregnancies and parturitions by healthy bitches, regularly vaccinated and dewormed before mating. During the second half of gestation, all bitches had been fed a pregnancy-specific commercial diet. The study was strictly conducted on normal puppies only, i.e. considered as conforming with their specific breed. All enrolled puppies showed normal development and weight, no malformations or physical defects, and their death had occurred suddenly without any disease interference on their weight gain and growth. To be eligible for the study they had to satisfy the following criteria: stillborn puppies, dying because of intra-partum asphyxia, born alive but dying within 1 hour after birth; puppies dying within their first week of age because of sudden death (i.e. at an interval between first symptoms and death shorter than 24 hours), caused by sudden septicemia, as evidenced by post mortem examination. From the time of their death, puppies were stored at 4°C for less than 12 hours and refrigerated during their transfer to the laboratory unit at Università degli Studi di Milano. Breed, gender, age and body weight were recorded before their storage at– 20°C.

### Measures

#### Anatomic measures and indices

**Fig 1:**
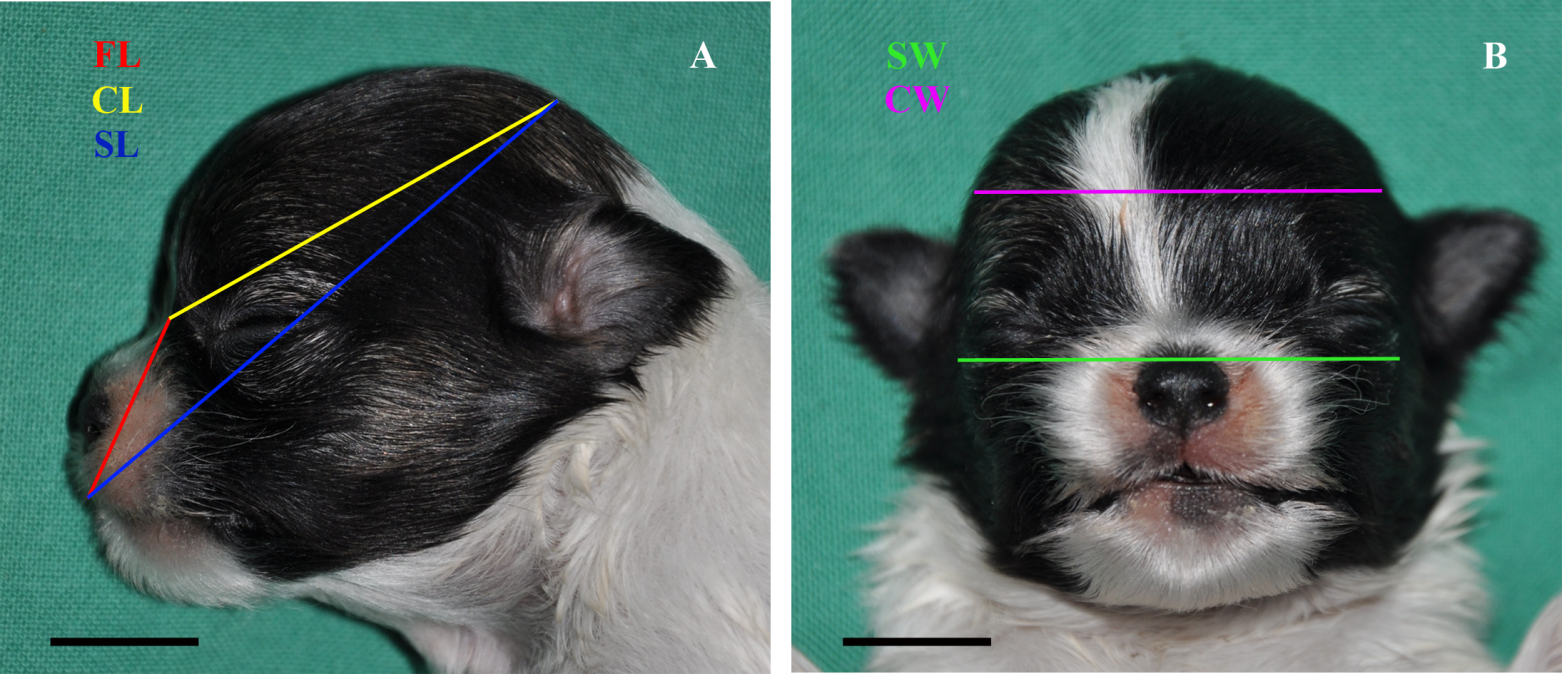
Anatomic linear measures. A: Facial Length (FL); Cranial Length (CL); Skull Length (SL); B: Skull Width (SW); Cranial Width (CW); Bar = 1 cm.

Consistent with literature [16], the following linear measures were obtained for each dog by a calliper: Cranial Length (CL), Cranial Width (CW), Skull Length (SL), Skull Width (SW) and Facial Length (FL). Every measure was blindly repeated three times on the whole head, accurately palpating its landmarks (Fig 1). The following indices were also calculated: Cranial Index (CI) and Skull Index (SI) [16] (Tables 1 and 2).

**Table 1:** Linear measures.

Linear measures	Landmarks
Skull Length (SL)^a^	from *Prosthion* to *Inion*
Cranial Length (CL)^a^	from *Inion* to *Nasion*
Cranial Length on LL view (CL-LL)^a^*	from *Inion* to the caudal edge of the fronto-nasal suture
Facial Length (FL)^a^	from *Nasion* to *Prosthion*
Facial Length on LL view (FL-LL)^a^*	from *Prosthion* to the caudal edge of the fronto-nasal suture
Cranial Width (CW)^a^	the most lateral points of the neurocranium
Skull Width (SW)^a^	the most lateral points of the zygomatic arch
Facial Length DV (FL-DV)^b^	from *Prosthion* to *Nasion*
Cranial Length DV (CL-DV)^b^	from *Nasion* to the caudal edge of the occipital condyle

^a^Evans and de Lahunta, 2013 [16]

^b^Koch et al., 2012 [15]

*modified measure (S1 Table).

**Table 2:** Indices.

Index	Formula
Cranial index (CI) ^a^	(CW x 100)/CL
Skull index (SI) ^a^	(SW x 100)/SL
S-index (S-I) ^b^	FL-DV/CL-DV
Facial index (FI) ^a^	(SW x 100)/FL

^a^Evans and de Lahunta, 2013 [16]

^b^Koch et al., 2012 [15].

#### Radiographic measures and indices

**Fig 2:**
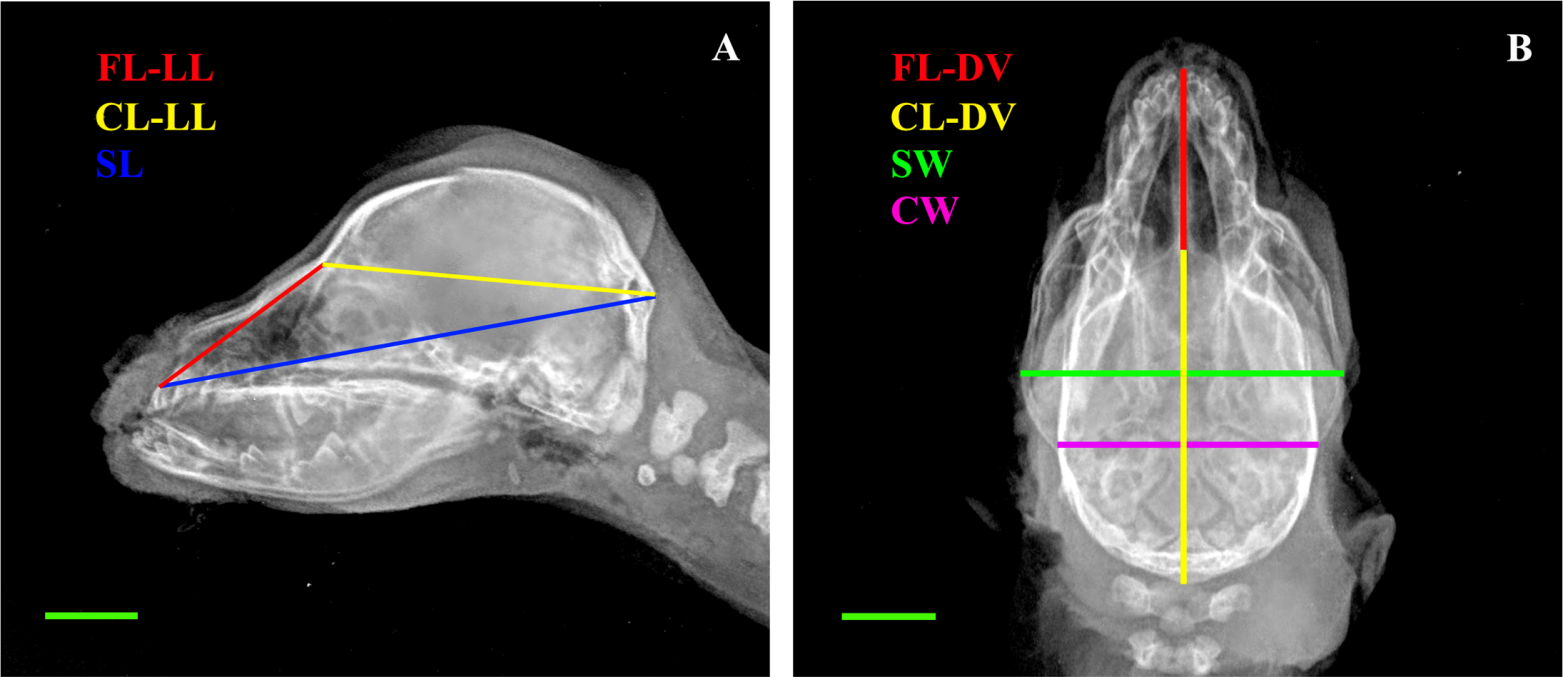
Radiographic linear measures. A: Latero-lateral view (LL): Facial Length (FL-LL); Cranial Length (CL-LL); Skull Length (SL); B: Dorso-ventral view (DV): Facial Length (FL-DV); Cranial Length (CL-DV); Skull Width (SW); Cranial Width (CW); Bar = 1 cm.

Radiographic exams were performed by a CR system (FCR Fuji Capsula X®) assembled with a radiological unit (ARCOM–Simply), using a 0.6 mm focal spot. The focal spot-film distance was 100 cm and no grid was employed. Latero-lateral (LL) and dorso-ventral (DV) views of the skull were obtained for each puppy. The images were stored in an Apple database and post-processing measures were performed by Osirix PRO^®^. Facial Length (FL-DV) and Cranial Length (CL-DV) were obtained on DV view [15]. Additional linear measures, extrapolated from the corresponding anatomic measures, were evaluated [16]. Some of them, Cranial Width (CW), Skull Width (SW) and Skull Length on LL view (SL-LL), were transferred unaltered. Others were modified, i.e. Cranial Length on LL view (CL-LL) was measured from *Inion* to the most caudal part of the fronto-nasal suture and Facial Length on LL projection (FL-LL) was measured from *Prosthion* to the most caudal part of the fronto-nasal suture (Fig 2). Every measure was blindly repeated three times. The S-index (S-I) was calculated according to literature [16]. Additional indices were extrapolated from the corresponding anatomic ones: Cranial Index (CI), Facial Index (FI) and Skull Index (SI) [16] (Tables 1 and 2).

The adopted terminology was chosen in accordance to the *Nomina Anatomica Veterinaria* (2012) and to the textbook “Miller's anatomy of the dog” [16].

### Statistical analysis

Repeatability of each measure taken in triple was evaluated by Friedman’s test and the mean value for each measurement was considered for further statistical analysis. Analysis of variance was performed on puppy groups according to the traditional craniometric categories (brachycephalic, mesaticephalic, dolichocephalic) to detect differences among the groups. Agreement between anatomical and radiographic linear measurements was evaluated by the graphical method of Bland-Altman plots and also bias between tests was calculated. Results are graphically reported indicating the average *versus* the difference between the couples of variables. Two confidence bands (generally 95%) delimit the cloud of points to evaluate the number of points falling into the bands space, thus indicating goodness of concordance between the two methods. Neural nets were used in the attempt to classify puppies belonging to unclassified breeds within the categories of brachycephalic, mesaticephalic or dolichocephalic. Standardized radiographic parameters were classified by cluster analysis after processing in an artificial neural network. The neural network was the unsupervised perceptron network, with a holdback value of 0.6 and three hidden nodes. Through the training set, the neural network can classify new cases based on the experience acquired. Analysis of variance was further performed after the new classification obtained by neural nets, as internal control. Statistical analysis was performed by the program JMP7.0 (SAS Inst., Inc., NC, USA) and the software XLstat for Windows platform.

## Results

**Fig 3:**
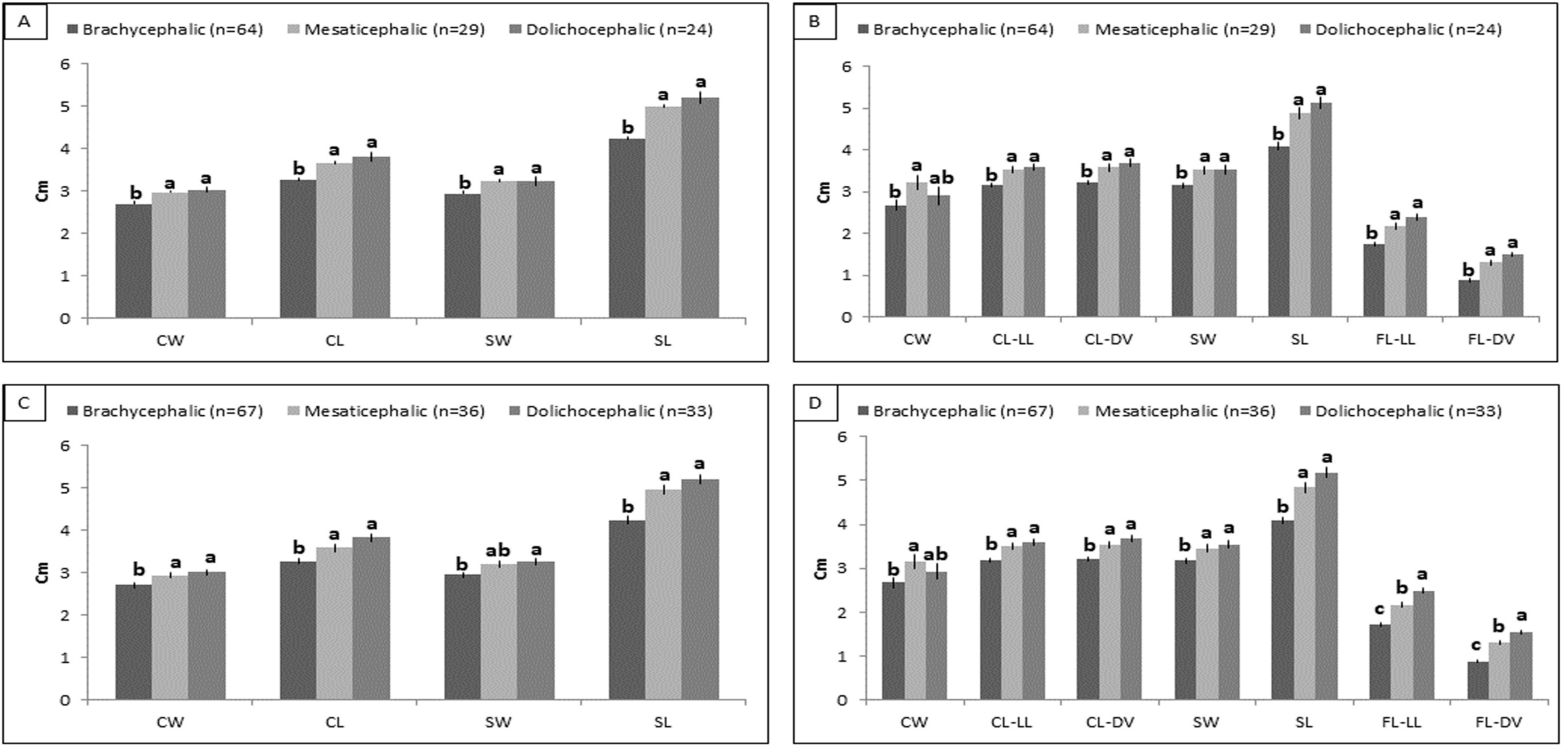
ANOVA for anatomic and radiographic linear measures pre- and post-neural net. Anatomic linear measures pre- (A) and post- (C) neural nets; Radiologic linear measures pre- (B) and post- (D) neural nets. Values (means±SEM) are expressed as cm. ^a-c^ Means with different letters within rows are significantly different (*p*<0,05). Cranial Width (CW); Cranial Length (CL); Skull Width (SW); Skull Length, (SL); Cranial Length LL (CL-LL); Cranial Length DV (CL-DV); Facial Length LL (FL-LL); Facial Length DV (FL-DV).

One hundred thirty-seven puppies (0–7 days) belonging to 33 different breeds met the inclusion criteria and were categorized according to literature. In case of discrepancies in the results derived from different studies, the cephalic index, when available from literature [26, 27, 33, 37] was employed to define the category for each breed, together with the results of previous studies [5–16]. Few breeds had never been included in any craniometric study and were considered unclassified: a) Dolichocephalic (n = 24): Afghan Hound (n = 5), Schnauzer (giant) (n = 5), English Setter (n = 4), German Shepherd (n = 3), Springer Spaniel (n = 3), Whippet (n = 1), Dachshund (n = 1), Hovawart (n = 1), Saint Bernard (n = 1); b) Mesaticephalic (n = 29): Labrador Retriever (n = 7), Leonberger (n = 5), Jack Russel Terrier (n = 5), Shar Pei (n = 3), Beagle (n = 2), American Cocker Spaniel (n = 2), Pinscher (n = 2), Alaskan Malamute (n = 1), Golden Retriever (n = 1), Border Collie (n = 1); c) Brachycephalic (n = 64): Chihuahua (n = 25), Bullmastiff (n = 13), English Bulldog (n = 9), Rottweiler (n = 8), Maltese (n = 4), Shih Tzu (n = 2), Boxer (n = 1), American Staffordshire Terrier (n = 1), Epagneul Breton (n = 1); d) Unclassified (n = 20): Poodle (toy) (n = 8), Maremma Sheepdog (n = 6), Jagd Terrier (n = 4), Bull Terrier (miniature) (n = 1), Belgian Shepherd (n = 1). Results of anatomic and radiographic linear measures are provided as supporting information (S3 Table and S4 Table, respectively). Results of the ANOVA performed on puppies classified as dolichocephalic, mesaticephalic and brachycephalic according to literature are shown in (Fig 3A and 3B) and Table 3.

**Table 3:** ANOVA for anatomic and radiographic indices pre- and post-neural nets (mean±SEM).

**Pre-** **Neural Nets**	**Indices**	**Brachycephalic** **(n = 64)**	**Mesaticephalic** **(n = 29)**	**Dolichocephalic** **(n = 24)**	***p***
**Anatomy**	**SI**	69.67 ±0.54^a^	64.36±0.69^b^	62.53±0.75^b^	***
	**CI**	84.26±2.05^a^	81.245±0.69^b^	80.56±0.76^b^	***
**Radiology**	**SI**	70.89±0.38^a^	66.33±0.52^b^	64.29±0.56^c^	***
	**CI**	84.26±2.05	89.56±2.77	81.24±2.99	
	**FI**	182.99±3.17	161.61±2.37^b^	146.04±2.55^c^	***
	**S-I**	0.26±0.01^c^	0.36±0.01^b^	0.41±0.01^a^	***
**Post-** **Neural Nets**	**Indices**	**Brachycephalic** **(n = 67)**	**Mesaticephalic** **(n = 36)**	**Dolichocephalic** **(n = 33)**	***p***
**Anatomy**	**SI**	69.50±0.54^a^	64.30±0.71^b^	62.90±0.73^b^	***
	**CI**	83.34±0.50^a^	82.02±0.73^b^	79.12±0.75^b^	***
**Radiology**	**SI**	71.12±0.35^a^	66.20±0.49^b^	63.88±0.50^c^	***
	**CI**	84.38±2.09	89.84±2.93	82.04±2.97	
	**FI**	186.31±1.61^a^	159.42±2.26^b^	143.96±2.30^c^	***
	**S-I**	0.27±0.01^c^	0.37±0.01^b^	0.42±0.01^a^	***

Values are expressed as means±SEM.

^a-c^ Means with different letters within rows are significantly different (*p*<0,05). Asterisks evidence the ANOVA significance (***p<0.01).

Skull index (SI); Cranial Index (CI); Facial Index (FI); S-Index (S-I).

**Fig 4:**
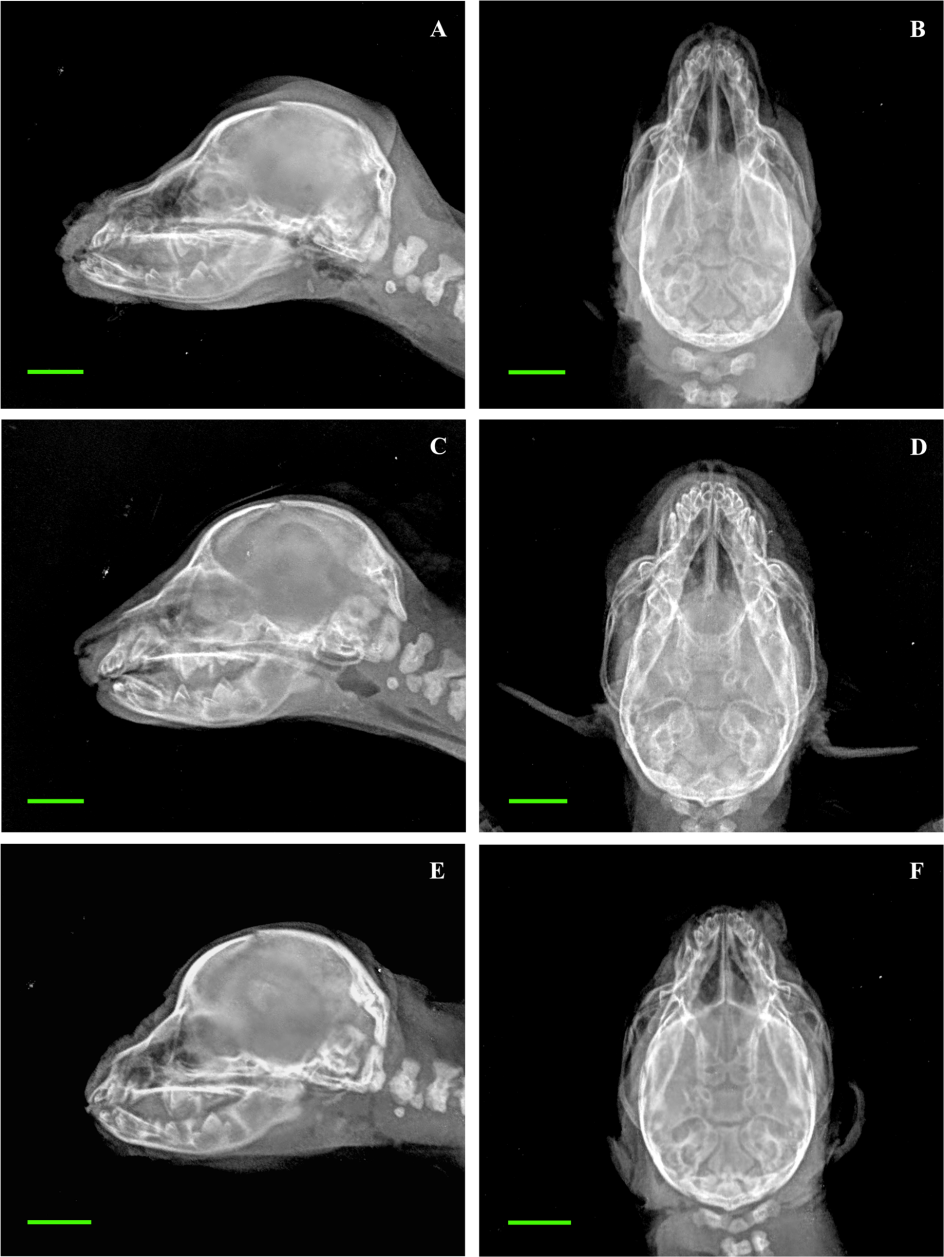
Radiographic exams depicting differences in skull shape among dolichocephalic, mesaticephalic and brachycephalic newborn puppies. Representative images of a dolichocephalic puppy (Afghan Hound A, B), a mesaticephalic puppy (Labrador Retriever C, D) and a brachycephalic puppy (Chihuahua E, F). A, C, E: Latero-lateral views; B, D, F: Dorso-ventral views. Bar = 1 cm.

All linear measures and anatomic indices significantly differed among brachycephalic and non-brachycephalic puppies. Only the radiographic CW identified dolichocephalic puppies as intermediate between brachycephalic and mesaticephalic ones. On the other hand, radiographic indices (with the exception of the CI) discriminate among the three categories (Fig 4).

**Fig 5:**
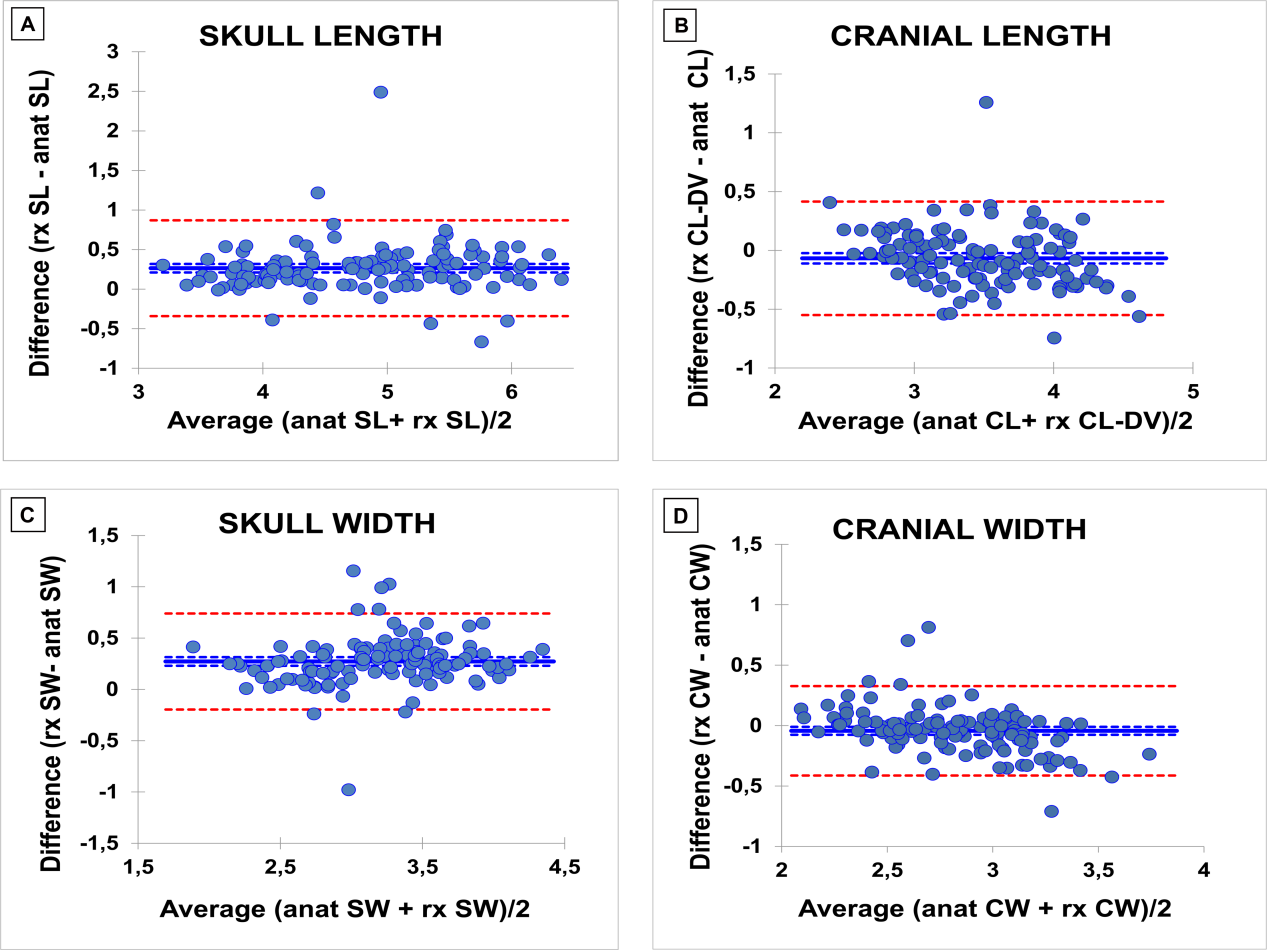
Bland–Altman difference plots to compare radiographic and anatomic measures. Differences between 2 values are plotted against the mean of the 2 values. The blue solid line represents the bias (mean difference) and the red dotted lines represent the 95% limits of agreement. A: Skull Length (SL); B: Cranial Length (CL); C: Skull Width (SW); D: Cranial Width (CW).

Bland-Altman plots for anatomic and radiographic linear measures indicate that a limited though unacceptable number of outliers is present for all measures. Graphs depict the bias, the bias 95% confidence interval and the 95% confidence interval for the data: CW -0.043±0.189; CL -0.067±0.246; SW 0.272±0.239; SL 0.265±0.309 (Fig 5).

Results from the new classification of puppies with neural nets indicate that 19/19 (100%) puppies belonging to 5 previously unclassified breeds were categorized as dolichocephalic (n = 9), mesaticephalic (n = 7) and brachycephalic (n = 3) with different probabilities (P between 0.66 and 0.95) (S2 Table). One puppy (Poodle toy) was excluded from the neural nets analysis due to missing radiographic data.

Results of the ANOVA performed including puppies newly classified with neural nets are shown in (Fig 3C and 3D) and Table 3: they confirm the results of the previous ANOVA for almost all parameters (*p*<0.05).

## Discussion

To the authors’ knowledge, this is the first multi-breed craniometric study on newborn puppies based on linear measures and indices. In fact, the present investigation provides new insights on the craniometry of newborn puppies aged 0–7 days belonging to 33 different breeds. The first aim of this work was to verify whether the craniometric differences that are typical of adult dogs (brachycephalic, mesaticephalic, dolichocephalic) are already present in newborn purebred puppies during the first week of age. Grouping puppies into these three categories allowed highlighting significant differences among them. Linear measures almost constantly identified two groups: brachycephalic *vs* non-brachycephalic morphotype (Fig 3), as previously described by Starck [35]. Anatomy and radiography provided contrasting results about the indices. While anatomic indices highlighted differences in skull conformation without a clear identification of three separate categories, almost all radiographic indices constantly distinguished among the three categories. The radiographic CI is the only index that displayed no differences among puppies (Table 3): this could be due to a quite uniform cranial shape among puppies, as supported by the anatomical corresponding index, which only isolates the brachycephalic morphotype. However, it should also be taken into account that radiographic landmarks (e.g. frontonasal suture) may be challenging to identify in newborns, as shown by their higher SEM. These results were not surprising: in fact, our study suggests that anatomic and radiographic methods cannot be used interchangeably when measuring puppies’ skulls. Being radiographic measures considered as invasive, we tried to provide a non-invasive method: despite a low bias, the differences in Bland-Altman plots (sometimes even higher than 1 cm) were considered unacceptably high for the purpose (Fig 5). This negative though expected result may be likely due to the presence of soft tissues that make radiographic and anatomic landmarks markedly different.

The last aim of this work was to classify as dolichocephalic, mesaticephalic or brachycephalic puppies belonging to previously uncategorized breeds using neural nets. Neural nets provided useful craniometric information, assigning 19/19 puppies (100%) to the three categories with different probability. Some of the skulls were classified with relatively low probability (e.g a Jagd Terrier was classified as dolichocephalic with P = 0,66, S2 Table): this could be due to a limited over-fitting effect of the neural network procedure and/or to the inner multivariate variability of each sample. Multivariate samples hide an intimate structure that a “classical” examination (as for the historical classification) cannot put on the surface. Moreover, it must be taken into account that growing animals are submitted to dramatic morpho-functional allometric changes [23], that in some animals can evolve more or less rapidly compared to others. After the new classification, an ANOVA test was repeated as internal control, including the newly classified puppies in their respective groups. The results largely confirmed the previously performed ANOVA: this was considered as a proof of the results of the neural nets, which were used for the first time in this study in attempt to craniometrically classify newborn puppies. Neural nets are bio-inspired computational models created to simulate the human brain data processing, consisting of networks of highly interconnected virtual neurons that can autonomously output decisions based on previously provided input information. Thus, neural nets are able to learn from past experience through a specific training process and provide outcome on new data based on such experience [38–40]. This learning ability makes them perfectly suitable for the solution of classification issues. As a basis, the large amount of radiographic data obtained from puppies belonging to classified breeds was used for the set-up in this study. However, growing animals cannot perfectly fit the static nature of neural nets: since the skull does not grow in all directions at the same time, the classification determined during the first week of age could be contradicted by what determined during other periods of their skull growth. For this reason, this method can represent a useful but not definitive tool to define puppies’ morphotype, that in our opinion could be more helpful in the categorization of adult dogs.

A few flaws are present in this study. A very small sample size available for some breeds (e.g. Belgian Shepherd, Saint Bernard and Hovawart) could have influenced its results, especially the ones obtained by the neural nets, which may not be fully representative for a larger population (breed). Unfortunately, uneven sampling is often intrinsic in cadaveric studies and hardly ever avoidable. However, the main purpose of the study, excluding the definition of breed standards for the puppies, was in favour of the choice to enrol all available puppies, irrespective of their number per breed. Increasing sample size and homogeneity could allow a better definition of breed-specific craniometry and establish breed standard references to evaluate skull development in newborn puppies. It could also help to define cut-off values to early recognise skull-shape measures linked to pathologies’ predisposition such as Chiari-like malformation [14, 41–43] and brachycephalic obstructive airways syndrome (BOAS) [44]. For this reason, more studies on puppies belonging to predisposed breeds could provide new clinical insights in dogs as well as in humans. A recent study of dog DNA revealed a genetic mutation linked to two brachycephalic breeds, suggesting that the craniofacial diversity of dogs could be useful to discover candidate genes involved in canine as well as human craniofacial anomalies [45]. Future perspectives also include the evaluation of adult dogs belonging to unclassified breeds, aiming to apply neural nets in a “craniometrically stable” sample.

## Conclusion

This study ascertained for the first time the skull morphometric differences among dolichocephalic, mesaticephalic and brachycephalic purebred puppies in their early neonatal period. Such differences were observed after both anatomic and radiologic evaluation, constantly isolating brachycephalic from non-brachycephalic puppies. Anatomic and radiologic measures, however, were not interchangeable. The investigation made it also possible to reliably classify 19 puppies belonging to 5 previously uncategorized breeds using the neural nets. Moreover, it suggested that canine cadavers can represent a valid alternative to *in vivo* animal models in the study of skeleton development, as previously demonstrated [36, 46].

## Supporting information

S1 Table Landmarks description [16]. (DOCX)

S2 Table Results of the neural nets. The second, third and fourth columns show the probability for each uncategorized puppy to be classified in the corresponding craniometric group. Results are indicated as the probability between 0–1. The highest probability is bold-typed.
(DOCX)

S3 Table Anatomic linear measures. Mean values, expressed in cm.
S = stillborn. M = male; F = female. In red, brachycephalic breeds; in blue, mesaticephalic breeds; in green, dolicocephalic breeds; in black, unclassified breeds. CW = Cranial Width, CL = Cranial Length, SW = Skull Width, SL = Skull Length, SI = Skull Index, CI = Cranial Index, MD = Missing Data.
(DOCX)

S4 Table Radiographic linear measures. Mean values, expressed in cm.
S = stillborn. M = male; F = female. In red, brachycephalic breeds; in blue, mesaticephalic breeds; in green, dolicocephalic breeds; in black, unclassified breeds. CL = Cranial Length, FL = Facial Length, FLDV = Facial Length on DV projection, CLDV = Cranial Length on DV projection, SL = Skull Length, CBL = Condylobasal length, SW = Skull Width, CW = Cranial Width, MD = Missing Data.
(DOCX)

## References

[1] Drake AG, Klingenberg CP. Large-scale diversification of skull shape in domestic dogs: disparity and modularity. Am Nat. 2010;175(3):289–301. doi: 10.1086/650372 20095825 10.1086/650372

[2] Saber AS, Gummow B. Skull morphometry of the lion (Panthera leo), dog (Canis lupus familiaris) and cat (Felis catus). Journal of Veterinary Anatomy. 2015;8(1):13–30.

[3] Getty R. Sisson and Grossman 's The Anatomy of the Domestic Animals Fifth Edition ed. Sisson S, Grossman JD, Getty R, editors. Philadelphia: W.B. Saunders Company; 1975. 2480 p.

[4] Dyce KM, Sack WO, Wensing CJG. Textbook of Veterinary Anatomy 4th Revised Edition ed. St Louis-Missouri: Sunders/Elsevier; 2010. 864 p.

[5] Ellenberger W, Baum H. Handbuch der vergleichenden Anatomie der Haustiere Berlin: Hirschwald, A; 1932. 1156 p.

[6] Stockard CR. The genetic and endocrinic basis for differences in form and behavior American Anatomy Memoir. 19. Philadelphia: Wistar Institute of Anatomy and Biology; 1941.

[7] Bourdelle E, Bressou C. Anatomie regionale des animaux domestiques. Paris1953.

[8] Seiferle E. [On the topography of the brain on long and short skulls in dog breeds]. Acta anatomica. 1966;63(3):346–62. 5918444

[9] Brehm H, Loeffler K, Komeyli H. [Skull forms in dogs]. Anatomia, histologia, embryologia. 1985;14(4):324–31. 2936276 10.1111/j.1439-0264.1985.tb00828.x

[10] Lignereux Y, Regodon S, Pavaux C. Cephalic typology in dogs Typologie cephalique canine. Revue de Medecine Veterinaire. 1991;142(6):469–80.

[11] Onar V, Ozcan S, Pazvant G. Skull typology of adult male Kangal dogs. Anatomia, histologia, embryologia. 2001;30(1):41–8. 11284162 10.1046/j.1439-0264.2001.00292.x

[12] Alpak H, Mutus R, Onar V. Correlation analysis of the skull and long bone measurements of the dog. Annals of anatomy = Anatomischer Anzeiger: official organ of the Anatomische Gesellschaft. 2004;186(4):323–30. 15481839 10.1016/S0940-9602(04)80050-5

[13] Gacsi M, McGreevy P, Kara E, Miklosi A. Effects of selection for cooperation and attention in dogs. Behav Brain Funct. 2009;5:31 doi: 10.1186/1744-9081-5-31 19630939 10.1186/1744-9081-5-31 PMC2731781

[14] Schmidt MJ, Neumann AC, Amort KH, Failing K, Kramer M. Cephalometric measurements and determination of general skull type of Cavalier King Charles Spaniels. Veterinary radiology & ultrasound: the official journal of the American College of Veterinary Radiology and the International Veterinary Radiology Association. 2011;52(4):436–40. 10.1111/j.1740-8261.2011.01825.x 21521397

[15] Koch DA, Wiestner T, Balli A, Montavon PM, Michel E, Scharf G, et al Proposal for a new radiological index to determine skull conformation in the dog. SAT, Schweizer Archiv fur Tierheilkunde. 2012;154(5):217–20. doi: 10.1024/0036-7281/a000331 22547337 10.1024/0036-7281/a000331

[16] Evans HE, De Lahunta A. Miller's Anatomy of the Dog Fourth edition ed. St Louis (MO) USA: Saunders/Elsevir; 2013. 872 p.

[17] Bannasch D, Young A, Myers J, Truve K, Dickinson P, Gregg J, et al Localization of canine brachycephaly using an across breed mapping approach. PloS one. 2010;5(3):e9632 doi: 10.1371/journal.pone.0009632 20224736 10.1371/journal.pone.0009632 PMC2835769

[18] Boyko AR, Quignon P, Li L, Schoenebeck JJ, Degenhardt JD, Lohmueller KE, et al A simple genetic architecture underlies morphological variation in dogs. PLoS Biol. 2010;8(8):e1000451 doi: 10.1371/journal.pbio.1000451 20711490 10.1371/journal.pbio.1000451 PMC2919785

[19] Shearin AL, Ostrander EA. Canine morphology: hunting for genes and tracking mutations. PLoS Biol. 2010;8(3):e1000310 doi: 10.1371/journal.pbio.1000310 20209140 10.1371/journal.pbio.1000310 PMC2830451

[20] Schoenebeck JJ, Ostrander EA. The genetics of canine skull shape variation. Genetics. 2013;193(2):317–25. doi: 10.1534/genetics.112.145284 23396475 10.1534/genetics.112.145284 PMC3567726

[21] Trouth CO, Winter S, Gupta KC, Millis RM, Holloway JA. Analysis of the sexual dimorphism in the basiocciptal portion of the dog's skull. Acta anatomica. 1977;98(4):469–73. 883490 10.1159/000144826

[22] Onar V. A morphometric study on the skull of the German shepherd dog (Alsatian). Anatomia Histologia Embryologia-Journal of Veterinary Medicine Series C-Zentralblatt Fur Veterinarmedizin Reihe C. 1999;28(4):253–6. 10.1046/j.1439-0264.1999.00202.x 10488631

[23] Onar V, Gunes H. On the variability of skull shape in German shepherd (Alsatian) puppies. Anatomical Record Part a-Discoveries in Molecular Cellular and Evolutionary Biology. 2003;272A(1):460–6. 10.1002/ar.a.10052 12704704

[24] Trangerud C, Grondalen J, Indrebo A, Tverdal A, Ropstad E, Moe L. A longitudinal study on growth and growth variables in dogs of four large breeds raised in domestic environments. Journal of Animal Science. 2007;85(1):76–83. doi: 10.2527/jas.2006-354 17179542 10.2527/jas.2006-354

[25] Sutter NB, Mosher DS, Gray MM, Ostrander EA. Morphometrics within dog breeds are highly reproducible and dispute Rensch's rule. Mammalian Genome. 2008;19(10/12):713–23. 19020935 10.1007/s00335-008-9153-6 PMC2748280

[26] McGreevy PD, Georgevsky D, Carrasco J, Valenzuela M, Duffy DL, Serpell JA. Dog behavior co-varies with height, bodyweight and skull shape. PloS one. 2013;8(12):e80529 doi: 10.1371/journal.pone.0080529 24358107 10.1371/journal.pone.0080529 PMC3864788

[27] Carrasco JJ, Georgevsky D, Valenzuela M, McGreevy PD. A pilot study of sexual dimorphism in the head morphology of domestic dogs. Journal of Veterinary Behavior: Clinical Applications and Research. 2014;9(1):43–6.

[28] Georgevsky D, Carrasco JJ, Valenzuela M, McGreevy PD. Domestic dog skull diversity across breeds, breed groupings, and genetic clusters. Journal of Veterinary Behavior: Clinical Applications and Research. 2014;9(5):228–34.

[29] Regodon S, Vivo JM, Franco A, Guillen MT, Robina A. Craniofacial angle in dolicho-, meso- and brachycephalic dogs: radiological determination and application. Annals of anatomy = Anatomischer Anzeiger: official organ of the Anatomische Gesellschaft. 1993;175(4):361–3. 8363043 10.1016/s0940-9602(11)80043-9

[30] McGreevy P, Grassi TD, Harman AM. A strong correlation exists between the distribution of retinal ganglion cells and nose length in the dog. Brain Behav Evol. 2004;63(1):13–22. doi: 10.1159/000073756 14673195 10.1159/000073756

[31] Helton WS. Cephalic index and perceived dog trainability. Behav Processes. 2009;82(3):355–8. doi: 10.1016/j.beproc.2009.08.004 19683035 10.1016/j.beproc.2009.08.004

[32] Stone HR, McGreevy PD, Starling MJ, Forkman B. Associations between Domestic-Dog Morphology and Behaviour Scores in the Dog Mentality Assessment. PloS one. 2016;11(2):e0149403 doi: 10.1371/journal.pone.0149403 26919495 10.1371/journal.pone.0149403 PMC4771026

[33] Teng KT, McGreevy PD, Toribio JA, Dhand NK. Trends in popularity of some morphological traits of purebred dogs in Australia. Canine Genet Epidemiol. 2016;3:2 doi: 10.1186/s40575-016-0032-2 27051522 10.1186/s40575-016-0032-2 PMC4820977

[34] Pilegaard AM, Berendt M, Holst P, Moller A, McEvoy FJ. Effect of Skull Type on the Relative Size of Cerebral Cortex and Lateral Ventricles in Dogs. Front Vet Sci. 2017;4:30 doi: 10.3389/fvets.2017.00030 28361057 10.3389/fvets.2017.00030 PMC5352664

[35] Starck D. Der Heutige Stand de fetalisationsproblems J Anim Breed Genet. 1962;77:129–55.

[36] Modina SC, Veronesi MC, Moioli M, Meloni T, Lodi G, Bronzo V, et al Small-sized newborn dogs skeletal development: radiologic, morphometric, and histological findings obtained from spontaneously dead animals. BMC veterinary research. 2017;13(1):175 doi: 10.1186/s12917-017-1092-6 28615055 10.1186/s12917-017-1092-6 PMC5471892

[37] Roberts T, McGreevy PD. Selection for breed-specific long-bodied phenotypes is associated with increased expression of canine hip dysplasia. Vet J. 2010;183(3):266–72. doi: 10.1016/j.tvjl.2009.11.005 19959383 10.1016/j.tvjl.2009.11.005

[38] Fogel GB. Computational intelligence approaches for pattern discovery in biological systems. Brief Bioinform. 2008;9(4):307–16. doi: 10.1093/bib/bbn021 18460474 10.1093/bib/bbn021

[39] Contri A, Zambelli D, Faustini M, Cunto M, Gloria A, Carluccio A. Artificial neural networks for the definition of kinetic subpopulations in electroejaculated and epididymal spermatozoa in the domestic cat. Reproduction. 2012;144(3):339–47. doi: 10.1530/REP-12-0125 22753767 10.1530/REP-12-0125

[40] Pouliakis A, Karakitsou E, Margari N, Bountris P, Haritou M, Panayiotides J, et al Artificial Neural Networks as Decision Support Tools in Cytopathology: Past, Present, and Future. Biomed Eng Comput Biol. 2016;7:1–18. 10.4137/BECB.S31601 PMC4760671 26917984

[41] Schmidt MJ, Amort KH, Failing K, Klingler M, Kramer M, Ondreka N. Comparison of the endocranial- and brain volumes in brachycephalic dogs, mesaticephalic dogs and Cavalier King Charles spaniels in relation to their body weight. Acta veterinaria Scandinavica. 2014;56(30):13 5 2014. 24886598 10.1186/1751-0147-56-30 PMC4038113

[42] Knowler SP, v/d Berg H, McFadyen A, La Ragione RM, Rusbridge C. Inheritance of Chiari-Like Malformation: Can a Mixed Breeding Reduce the Risk of Syringomyelia? PloS one. 2016;11(3):e0151280 doi: 10.1371/journal.pone.0151280 27008271 10.1371/journal.pone.0151280 PMC4805231

[43] Cross CL, McFadyen AK, Jovanovik J, Tauro A, Driver CJ, Fitzpatrick N, et al. Forebrain conformation changes in Chiari-like malformation. BSAVA Congress 2016 Proceedings, 7–10 April 2016, Birmingham, UK. 2016:542–3.

[44] Meola SD. Brachycephalic airway syndrome. Topics in Companion Animal Medicine. 2013;28(3):91–6. doi: 10.1053/j.tcam.2013.06.004 24182996 10.1053/j.tcam.2013.06.004

[45] Marchant TW, Johnson EJ, McTeir L, Johnson CI, Gow A, Liuti T, et al Canine Brachycephaly Is Associated with a Retrotransposon-Mediated Missplicing of SMOC2. Curr Biol. 2017;27(11):1573–84 e6. doi: 10.1016/j.cub.2017.04.057 28552356 10.1016/j.cub.2017.04.057 PMC5462623

[46] Di Giancamillo A, Andreis ME, Taini P, Veronesi MC, Di Giancamillo M, Modina SC. Cartilage canals in newborn dogs: histochemical and immunohistochemical findings. Eur J Histochem. 2016;60(3):2701 doi: 10.4081/ejh.2016.2701 27734993 10.4081/ejh.2016.2701 PMC5062639

