# Supplementary material for: Novel contributions in canine craniometry: Anatomic and radiographic measurements in newborn puppies
Source: PLoS One. 2018 May 8;13(5):e0196959. doi: 10.1371/journal.pone.0196959 (PMC5940217; doi:10.1371/journal.pone.0196959)
Supplement: S1 Table — (DOCX) [file pone.0196959.s001.docx]

| **Landmarks** | **Description** |
| --- | --- |
| **Inion** | The central surface point on the external occipital protuberance |
| **Nasion** | junction on the median plane of the right and left naso-frontal sutures |
| **Prosthion** | anterior end of the interincisive suture (located between the roots of the upper central incisor teeth) |
| **Euryon** | the most lateral point of the brain case |
| **Zygion** | the most lateral point of the zygomatic arch |
