# Supplementary material for: Novel contributions in canine craniometry: Anatomic and radiographic measurements in newborn puppies
Source: PLoS One. 2018 May 8;13(5):e0196959. doi: 10.1371/journal.pone.0196959 (PMC5940217; doi:10.1371/journal.pone.0196959)
Supplement: S2 Table — The second, third and fourth columns show the probability for each uncategorized puppy to be classified in the corresponding craniometric group. Results are indicated as the probability between 0–1. The highest probability is bold-typed. (DOCX) [file pone.0196959.s002.docx]

| **Breed** | **Brachycephalic** | **Mesaticephalic** | **Dolichocephalic** |
| --- | --- | --- | --- |
| Poodle (toy) | 0,068 | **0,895** | 0,037 |
| Poodle (toy) | 0,060 | **0,895** | 0,045 |
| Poodle (toy) | 0,066 | **0,894** | 0,040 |
| Poodle (toy) | 0,067 | **0,895** | 0,038 |
| Poodle (toy) | **0,948** | 0,033 | 0,019 |
| Poodle (toy) | 0,020 | **0,895** | 0,085 |
| Poodle (toy) | 0,084 | **0,872** | 0,044 |
| Bull Terrier (mini) | 0,031 | 0,092 | **0,877** |
| Jagd Terrier | 0,079 | 0,259 | **0,662** |
| Jagd Terrier | 0,023 | 0,076 | **0,901** |
| Jagd Terrier | 0,037 | 0,073 | **0,890** |
| Jagd Terrier | 0,072 | 0,119 | **0,809** |
| Maremma Sheepdog | **0,940** | 0,050 | 0,010 |
| Maremma Sheepdog | 0,047 | 0,089 | **0,864** |
| Maremma Sheepdog | 0,020 | **0,758** | 0,222 |
| Maremma Sheepdog | 0,029 | 0,092 | **0,879** |
| Maremma Sheepdog | 0,053 | 0,063 | **0,884** |
| Maremma Sheepdog | 0,036 | 0,064 | **0,900** |
| Belgian Shepherd | **0,951** | 0,039 | 0,010 |
