# Supplementary material for: Novel contributions in canine craniometry: Anatomic and radiographic measurements in newborn puppies
Source: PLoS One. 2018 May 8;13(5):e0196959. doi: 10.1371/journal.pone.0196959 (PMC5940217; doi:10.1371/journal.pone.0196959)
Supplement: S3 Table — Mean values, expressed in cm. S = stillborn. M = male; F = female. In red, brachycephalic breeds; in blue, mesaticephalic breeds; in green, dolicocephalic breeds; in black, unclassified breeds. CW = Cranial Width, CL = Cranial Length, SW = Skull Width, SL = Skull Length, SI = Skull Index, CI = Cranial Index, MD = Missing Data. (DOCX) [file pone.0196959.s003.docx]

| **Age (days)** | **Breed** | **Gender** | **CW** | **CL** | **SW** | **SL** |
| --- | --- | --- | --- | --- | --- | --- |
| 0 | Eng. Bulldog | M | 2,96 | 3,59 | 3,46 | 4,44 |
| 0 | Eng. Bulldog | M | 2,93 | 3,24 | 3,25 | 4,25 |
| 0 | Eng. Bulldog | M | 2,77 | 3,44 | 3,16 | 4,04 |
| 0 | Eng. Bulldog | M | 2,81 | 3,35 | 3,04 | 4,01 |
| 0 | Eng. Bulldog | M | 3,07 | 3,44 | 3,53 | 4,13 |
| 0 | Eng. Bulldog | F | 2,99 | 3,32 | 3,41 | 4,21 |
| 0 | Eng. Bulldog | F | 2,91 | 3,13 | 3,06 | 4,23 |
| S | Bullmastiff | M | 2,29 | 2,89 | 2,75 | 3,70 |
| S | Bullmastiff | M | 2,92 | 3,53 | 3,00 | 5,00 |
| S | Bullmastiff | F | 2,63 | 3,35 | 2,90 | 4,19 |
| S | Bullmastiff | M | 2,57 | 3,67 | 3,06 | 4,82 |
| S | Bullmastiff | M | 2,13 | 2,96 | 2,63 | 3,93 |
| S | Chihuahua | M | 2,28 | 2,81 | 2,46 | 3,73 |
| S | Chihuahua | F | 2,71 | 2,75 | 2,91 | 3,76 |
| S | Chihuahua | M | 2,63 | 2,87 | 2,73 | 3,83 |
| S | Chihuahua | F | 2,23 | 2,83 | 2,29 | 3,43 |
| S | Chihuahua | F | 2,21 | 2,67 | 2,30 | 3,67 |
| S | Chihuahua | F | 2,23 | 2,72 | 2,29 | 3,43 |
| S | Chihuahua | M | 2,57 | 3,03 | 2,73 | 3,90 |
| S | Chihuahua | F | 2,59 | 2,90 | 2,67 | 3,80 |
| S | Chihuahua | F | MD | MD | MD | MD |
| S | Chihuahua | F | MD | MD | MD | MD |
| S | Rottweiler | M | 3,04 | 3,88 | 3,29 | 5,09 |
| S | Rottweiler | F | 3,00 | 3,39 | 3,30 | 4,70 |
| S | Rottweiler | F | 2,56 | 2,97 | 2,63 | 4,19 |
| S | Rottweiler | F | 2,39 | 3,09 | 2,52 | 4,05 |
| S | Rottweiler | F | 3,02 | 3,69 | 3,27 | 5,07 |
| S | Rottweiler | F | 3,20 | 3,80 | 3,52 | 5,21 |
| S | Rottweiler | F | 3,41 | 4,36 | 3,60 | 5,44 |
| 0 | Bullmastiff | M | 2,81 | 3,47 | 3,07 | 4,55 |
| 0 | Bullmastiff | M | 2,58 | 3,47 | 2,98 | 4,58 |
| 0 | Bullmastiff | M | 2,31 | 3,22 | 2,66 | 3,97 |
| 0 | Bullmastiff | F | 2,51 | 3,16 | 2,80 | 4,07 |
| 0 | Chihuahua | M | 2,51 | 3,02 | 2,60 | 3,60 |
| 0 | Chihuahua | M | 2,39 | 2,73 | 2,37 | 3,65 |
| 0 | Chihuahua | M | MD | MD | MD | MD |
| 0 | Chihuahua | F | 2,69 | 3,07 | 2,78 | 3,96 |
| 0 | Chihuahua | F | 2,64 | 2,98 | 2,62 | 3,94 |
| 0 | Chihuahua | M | 2,67 | 3,21 | 2,86 | 4,27 |
| S | Bullmastiff | F | 3,60 | 4,29 | 4,10 | 6,00 |
| S | Bullmastiff | F | 3,26 | 4,20 | 4,01 | 5,56 |
| S | Bullmastiff | M | 3,44 | 4,08 | 3,76 | 5,46 |
| S | Bullmastiff | M | 2,47 | 3,44 | 2,72 | 4,16 |
| 5 | Amstaff | F | 3,13 | 3,42 | 3,49 | 4,77 |
| 4 | Chihuahua | F | 2,87 | 3,08 | 2,66 | 3,96 |
| 4 | Chihuahua | M | 2,63 | 3,13 | 2,78 | 3,93 |
| 0 | Chihuahua | F | 2,52 | 2,98 | 2,54 | 3,81 |
| 0 | Chihuahua | F | MD | MD | MD | MD |
| 4 | Chihuahua | F | MD | MD | MD | MD |
| 3 | Maltese | F | 2,27 | 2,63 | 2,02 | 3,43 |
| 0 | English Bulldog | F | 3,01 | 3,52 | 3,41 | 4,24 |
| 0 | Boxer | M | 3,12 | 4,32 | 3,90 | 5,55 |
| 0 | Maltese | F | 2,07 | 2,58 | 2,07 | 3,37 |
| 1 | Chihuahua | M | 2,20 | 2,53 | 2,26 | 3,50 |
| 2 | Chihuahua | F | 2,02 | 2,19 | 1,68 | 3,04 |
| 2 | Rottweiler | M | 3,40 | 4,05 | 3,61 | 5,37 |
| 3 | Maltese | M | MD | MD | MD | MD |
| 4 | Epagneul Breton | F | 2,80 | 3,26 | 2,93 | 4,53 |
| 3 | Chihuahua | M | 2,55 | 2,69 | 3,47 | 3,36 |
| 3 | English Bulldog | M | 3,03 | 3,74 | 3,54 | 4,68 |
| 5 | Maltese | F | 2,58 | MD | MD | 3,64 |
| 5 | Shih Tzu | M | 2,46 | 3,10 | 2,64 | 3,68 |
| 5 | Shih Tzu | M | 2,71 | 3,20 | 2,97 | 3,59 |
| 7 | Boxer | M | 3,87 | 5,13 | 4,88 | 7,10 |
| 7 | Chihuahua | F | 2,33 | MD | MD | 4,37 |
| S | Shar Pei | M | 3,05 | 3,92 | 3,48 | 5,23 |
| S | Shar Pei | M | 3,09 | 3,78 | 3,40 | 5,05 |
| S | Beagle | M | 3,14 | 3,77 | 3,29 | 4,87 |
| S | Beagle | M | 3,03 | 3,76 | 3,32 | 4,93 |
| S | Border Collie | F | 3,30 | 3,98 | 3,42 | 5,31 |
| S | Jack Russell T. | F | 2,65 | 3,12 | 2,79 | 4,62 |
| S | Jack Russell T. | M | 2,63 | 3,55 | 2,82 | 4,43 |
| S | Jack Russell T. | F | 2,19 | 2,81 | 2,23 | 3,76 |
| S | Jack Russell T. | M | 2,58 | 2,95 | 2,82 | 3,95 |
| S | Jack Russell T. | F | 2,80 | 3,12 | 2,94 | 4,25 |
| S | Pinscher | F | 2,27 | 2,41 | 2,11 | 3,65 |
| 0 | Shar Pei | F | 2,95 | 3,35 | 3,24 | 4,91 |
| 0 | American Cocker Sp. | M | 3,23 | 3,76 | 3,40 | 5,48 |
| 4 | Labrador R. | M | 2,60 | 3,27 | 2,80 | 4,83 |
| 4 | Labrador R. | M | 2,84 | 3,55 | 3,00 | 4,73 |
| 4 | Golden R. | F | 3,63 | 3,97 | 3,87 | 6,17 |
| 3 | Labrador R. | M | 2,87 | 4,00 | 3,30 | 5,27 |
| 3 | Labrador R. | M | 2,87 | 3,78 | 3,23 | 5,13 |
| 4 | Alaskan Malamute | M | 3,38 | 4,53 | 3,98 | 5,93 |
| 3 | Labrador R. | F | 3,13 | 3,72 | 3,46 | 5,18 |
| 3 | Labrador R. | M | 3,17 | 3,93 | 3,62 | 5,65 |
| 3 | Labrador R. | M | 3,02 | 3,72 | 3,61 | 5,34 |
| 3 | Leonberger | F | 3,34 | 4,08 | 3,52 | 5,79 |
| 3 | Leonberger | M | 3,25 | 4,44 | 3,66 | 5,72 |
| 4 | Leonberger | M | 3,37 | 4,06 | 3,65 | 5,41 |
| 4 | American Cocker Sp. | M | 2,87 | 3,52 | 3,17 | 4,59 |
| 4 | Pinscher | F | 2,26 | 2,87 | 2,35 | 3,79 |
| 5 | Leonberger | M | 3,84 | 4,67 | 4,27 | 6,98 |
| 7 | Leonberger | M | 3,13 | 4,38 | 3,41 | 5,57 |
| 7 | Leonberger | M | 3,20 | 3,77 | 3,63 | 5,10 |
| S | English Setter | F | 3,16 | 4,22 | 3,45 | 5,60 |
| S | Springer Sp. | M | 3,24 | 3,20 | 3,50 | 4,88 |
| S | Springer Sp. | M | 2,83 | 3,83 | 2,87 | 4,91 |
| S | Springer Sp. | F | 3,21 | 3,64 | 3,49 | 4,75 |
| 0 | Dachshund | F | 2,62 | 2,95 | 2,48 | 3,73 |
| 0 | Whippet | F | 2,87 | 3,56 | 2,89 | 4,71 |
| 3 | Afghan Hound | F | 2,79 | 3,55 | 3,03 | 5,29 |
| 4 | Afghan Hound | M | 2,82 | 3,83 | 3,10 | 5,13 |
| 4 | Afghan Hound | M | 3,07 | 4,02 | 3,40 | 5,66 |
| 3 | Afghan Hound | F | 2,97 | 4,20 | 3,18 | 5,84 |
| 3 | Afghan Hound | F | 3,05 | 4,20 | 3,21 | 5,73 |
| 3 | German Shepherd | M | 3,37 | 4,30 | 3,92 | 5,93 |
| 4 | German Shepherd | F | 2,87 | 3,73 | 3,21 | 4,98 |
| 3 | German Shepherd | F | 3,06 | 3,97 | 3,30 | 5,45 |
| 4 | Hovavart | F | 3,04 | 4,15 | 3,28 | 5,62 |
| 3 | Schnauzer giant | F | MD | MD | MD | MD |
| 4 | Schnauzer giant | F | 3,02 | 3,78 | 3,15 | 5,19 |
| 3 | Schnauzer giant | F | 3,08 | 3,82 | 3,45 | 5,58 |
| 4 | Schnauzer giant | M | 2,95 | 3,83 | 3,17 | 5,12 |
| 3 | Schnauzer giant | F | 3,04 | 4,06 | 3,29 | 5,17 |
| 4 | English Setter | F | 2,73 | 3,12 | 2,91 | 4,59 |
| 4 | English Setter | F | 3,33 | 3,82 | 3,52 | 5,23 |
| 4 | English Setter | M | 2,82 | 3,46 | 2,94 | 4,66 |
| 5 | Saint Bernard | F | 3,86 | 4,89 | 4,15 | 6,34 |
| S | Jagd T. | M | 2,89 | 3,56 | 3,17 | 4,75 |
| S | Jagd T. | M | 2,69 | 3,48 | 2,90 | 4,84 |
| S | Jagd T. | M | 2,83 | 3,61 | 3,08 | 4,69 |
| S | Jagd T. | M | 2,69 | 3,53 | 2,96 | 4,71 |
| S | Maremma Sheepdog | F | 3,11 | 3,62 | 3,31 | 4,81 |
| 3 | Maremma Sheepdog | M | 3,78 | 4,73 | 3,97 | 6,08 |
| 3 | Maremma Sheepdog | F | 3,45 | 4,54 | 3,86 | 6,12 |
| 4 | Maremma Sheepdog | M | 3,52 | 4,35 | 3,83 | 6,09 |
| 2 | Belgian Shepherd | M | MD | MD | MD | MD |
| 7 | Maremma Sheepdog | F | 3,18 | 3,97 | 3,52 | 5,39 |
| 7 | Maremma Sheepdog | F | 3,18 | 4,21 | 3,54 | 5,88 |
| 0 | Poodle toy | F | 2,35 | 2,87 | 2,44 | 3,99 |
| 2 | Poodle toy | M | 2,50 | 3,01 | 2,66 | 4,08 |
| 2 | Poodle toy | M | 2,53 | 2,94 | 2,61 | 3,94 |
| 3 | Poodle toy | F | 2,43 | 2,80 | 2,50 | 4,00 |
| 3 | Poodle toy | F | 2,27 | 2,66 | 2,31 | 3,81 |
| 4 | Poodle toy | F | 2,52 | 3,23 | 2,72 | 4,25 |
| 5 | Bull T. mini | M | 2,74 | 3,80 | 2,87 | 5,05 |
| 8 | Poodle toy | F | 2,38 | 2,80 | 2,42 | 3,96 |
| 8 | Poodle toy | M | 2,25 | 2,82 | 2,44 | 3,83 |
