# Supplementary material for: Novel contributions in canine craniometry: Anatomic and radiographic measurements in newborn puppies
Source: PLoS One. 2018 May 8;13(5):e0196959. doi: 10.1371/journal.pone.0196959 (PMC5940217; doi:10.1371/journal.pone.0196959)
Supplement: S4 Table — Mean values, expressed in cm. S = stillborn. M = male; F = female. In red, brachycephalic breeds; in blue, mesaticephalic breeds; in green, dolicocephalic breeds; in black, unclassified breeds. CL = Cranial Length, FL = Facial Length, FLDV = Facial Length on DV projection, CLDV = Cranial Length on DV projection, SL = Skull Length, CBL = Condylobasal length, SW = Skull Width, CW = Cranial Width, MD = Missing Data. (DOCX) [file pone.0196959.s004.docx]

| **Age (days)** | **Breed** | **Gender** | **CL** | **FL** | **FLDV** | **CLDV** | **SL** | **CBL** | **SW** | **CW** |
| --- | --- | --- | --- | --- | --- | --- | --- | --- | --- | --- |
| 0 | Eng. Bulldog | M | 3,30 | 1,56 | 0,67 | 3,35 | 4,32 | 4,03 | 3,69 | 3,00 |
| 0 | Eng. Bulldog | M | 3,27 | 1,82 | 0,79 | 3,14 | 4,36 | 3,91 | 3,57 | 2,99 |
| 0 | Eng. Bulldog | M | 3,30 | 1,54 | 0,94 | 3,13 | 4,24 | 4,08 | 3,55 | 3,03 |
| 0 | Eng. Bulldog | M | 3,15 | 1,70 | 0,79 | 3,01 | 4,35 | 3,76 | 3,33 | 2,80 |
| 0 | Eng. Bulldog | M | 3,32 | 1,42 | 0,56 | 3,41 | 4,26 | 3,95 | 3,58 | 2,97 |
| 0 | Eng. Bulldog | F | 3,24 | 1,56 | 1,03 | 3,09 | 4,37 | 4,14 | 3,50 | 3,04 |
| 0 | Eng. Bulldog | F | 3,25 | 1,64 | 0,79 | 3,09 | 4,35 | 3,88 | 3,63 | 2,82 |
| S | Bullmastiff | M | 3,02 | 3,86 | 1,59 | 4,15 | 6,19 | 5,71 | 3,78 | 3,10 |
| S | Bullmastiff | M | 3,47 | 1,87 | 1,22 | 3,41 | 4,89 | 4,61 | 3,47 | 2,51 |
| S | Bullmastiff | F | 3,26 | 2,05 | 1,04 | 3,27 | 4,59 | 4,30 | 3,31 | 2,45 |
| S | Bullmastiff | M | 3,56 | 1,73 | 0,76 | 3,58 | 4,82 | 4,35 | 3,46 | 2,47 |
| S | Bullmastiff | M | 3,12 | 1,81 | 0,86 | 2,98 | 4,29 | 3,85 | 3,02 | 2,30 |
| S | Chihuahua | M | 2,72 | 1,48 | 0,74 | 2,76 | 3,79 | 3,50 | 2,51 | 2,32 |
| S | Chihuahua | F | 2,84 | 1,53 | 0,71 | 2,95 | 3,95 | 3,67 | 2,97 | 2,61 |
| S | Chihuahua | M | 2,85 | 1,48 | 0,69 | 2,92 | 3,91 | 3,62 | 2,75 | 2,47 |
| S | Chihuahua | F | 2,90 | 1,46 | 0,68 | 3,05 | 3,97 | 3,73 | 2,71 | 2,60 |
| S | Chihuahua | F | 2,78 | 1,46 | 0,62 | 2,86 | 3,69 | 3,48 | 2,53 | 2,28 |
| S | Chihuahua | F | 2,74 | 1,24 | 0,45 | 2,87 | 3,60 | 3,31 | 2,52 | 2,38 |
| S | Chihuahua | M | 2,80 | 1,55 | 0,75 | 3,05 | 3,99 | 3,80 | 2,75 | 2,53 |
| S | Chihuahua | F | 2,89 | 1,45 | 0,60 | 3,03 | 3,93 | 3,62 | 2,73 | 2,57 |
| S | Chihuahua | F | 2,81 | 1,47 | MD | MD | 3,88 | MD | MD | MD |
| S | Chihuahua | F | 2,70 | 1,66 | 0,96 | 2,77 | 3,96 | 3,73 | 2,76 | 2,21 |
| S | Rottweiler | M | 3,68 | 2,11 | 1,05 | 3,81 | 5,24 | 4,80 | 3,54 | 3,13 |
| S | Rottweiler | F | 3,69 | 2,21 | 1,05 | 3,71 | 5,22 | 4,76 | 3,57 | 2,75 |
| S | Rottweiler | F | 3,31 | 1,58 | 0,76 | 3,31 | 4,38 | 4,08 | 2,97 | 2,73 |
| S | Rottweiler | F | 3,18 | 1,71 | 0,74 | 3,26 | 4,31 | 3,98 | 2,94 | 2,73 |
| S | Rottweiler | F | 3,73 | 1,97 | 0,80 | 4,02 | 5,19 | 4,82 | 3,64 | 3,15 |
| S | Rottweiler | F | 3,87 | 1,95 | 0,86 | 4,03 | 5,26 | 4,90 | 3,76 | 3,24 |
| S | Rottweiler | F | 4,04 | 2,45 | 1,34 | 4,12 | 5,94 | 5,46 | 4,25 | 3,42 |
| 0 | Bullmastiff | M | 3,36 | 2,16 | 1,12 | 3,32 | 4,83 | 4,42 | 3,39 | 2,54 |
| 0 | Bullmastiff | M | 3,48 | 1,92 | 0,96 | 3,45 | 4,92 | 4,43 | 3,62 | 2,65 |
| 0 | Bullmastiff | M | 3,20 | 1,85 | 0,98 | 3,27 | 4,57 | 4,26 | 3,43 | 2,54 |
| 0 | Bullmastiff | F | 3,09 | 1,90 | 0,93 | 3,24 | 4,62 | 4,21 | 3,24 | 2,45 |
| 0 | Chihuahua | M | 2,88 | 1,70 | 0,82 | 2,93 | 4,08 | 3,79 | 2,80 | 2,52 |
| 0 | Chihuahua | M | 2,83 | 1,51 | 0,66 | 2,84 | 3,85 | 3,49 | 2,65 | 2,34 |
| 0 | Chihuahua | M | 2,99 | 1,56 | 0,68 | 2,98 | 3,99 | 3,64 | 2,72 | 2,74 |
| 0 | Chihuahua | F | 2,91 | 1,91 | 0,75 | 3,11 | 4,24 | 3,90 | 2,94 | 2,67 |
| 0 | Chihuahua | F | 2,94 | 1,71 | 0,75 | 3,06 | 4,19 | 3,78 | 2,80 | 2,59 |
| 0 | Chihuahua | M | 3,02 | 1,42 | 0,51 | 3,03 | 3,88 | 3,53 | 2,62 | 2,85 |
| S | Bullmastiff | F | 4,18 | 2,45 | 1,72 | 3,98 | 6,12 | 5,78 | 4,41 | 3,23 |
| S | Bullmastiff | F | 3,94 | 2,68 | 1,36 | 4,12 | 5,97 | 5,47 | 4,20 | 3,05 |
| S | Bullmastiff | M | 3,92 | 2,61 | 1,19 | 4,14 | 5,94 | 5,33 | 4,10 | 3,10 |
| S | Bullmastiff | M | 3,34 | 1,82 | 1,49 | 3,26 | 4,98 | 4,75 | 3,71 | 2,47 |
| 5 | Amstaff | F | 3,57 | 2,14 | 1,49 | 3,42 | 5,20 | 4,92 | 3,74 | 3,17 |
| 4 | Chihuahua | F | 3,11 | 1,18 | 0,70 | 2,88 | 4,07 | 3,61 | 2,97 | 2,82 |
| 4 | Chihuahua | M | 3,00 | 1,43 | 0,69 | 2,87 | 4,12 | 3,59 | 2,96 | 2,60 |
| 0 | Chihuahua | F | 2,85 | 1,64 | 0,71 | 2,78 | 3,81 | 3,50 | 2,63 | 2,48 |
| 0 | Chihuahua | F | 3,43 | 1,87 | 1,34 | 3,29 | 4,97 | 4,62 | 3,46 | 2,80 |
| 4 | Chihuahua | F | 2,90 | 1,63 | 0,63 | 3,00 | 4,04 | 3,62 | 2,76 | 2,29 |
| 3 | Maltese | F | 2,55 | 1,28 | MD | MD | 3,53 | MD | 2,27 | 2,27 |
| 0 | Eng. Bulldog | F | 3,22 | 1,94 | MD | MD | 4,57 | MD | 3,76 | 2,98 |
| 0 | Boxer | M | 4,02 | 2,18 | 1,53 | 4,01 | 5,57 | 5,54 | 4,08 | 3,08 |
| 0 | Maltese | F | 2,53 | 1,46 | MD | 2,55 | 3,74 | 3,38 | 2,32 | 2,14 |
| 1 | Chihuahua | M | 2,57 | 1,50 | 0,56 | 2,71 | 3,66 | 3,28 | 2,27 | 2,15 |
| 2 | Chihuahua | F | 2,56 | 1,17 | 0,38 | 2,60 | 3,35 | 2,96 | 2,09 | 2,16 |
| 2 | Rottweiler | M | 3,82 | 2,20 | 1,07 | 4,04 | 5,51 | 5,09 | 3,73 | 3,13 |
| 3 | Maltese | M | 2,55 | 1,25 | 0,71 | 2,49 | 3,49 | 3,20 | 2,20 | 1,98 |
| 4 | Epagneul Breton | F | 3,23 | 2,06 | 1,04 | 3,38 | 4,86 | 4,45 | 3,22 | 2,73 |
| 3 | Chihuahua | M | 2,68 | 1,18 | 0,49 | 2,67 | 3,41 | 3,15 | 2,49 | 2,56 |
| 3 | Eng. Bulldog | M | 3,46 | 1,72 | 1,11 | 3,37 | 4,73 | 4,47 | 3,70 | 3,09 |
| 5 | Maltese | F | 2,83 | 1,45 | 0,75 | 2,78 | 3,91 | 3,52 | 2,68 | 2,54 |
| 5 | Shih Tzu | M | 2,98 | 1,38 | 0,57 | 3,02 | 3,99 | 3,59 | 2,81 | 2,34 |
| 5 | Shih Tzu | M | 2,98 | 1,57 | 0,70 | 3,02 | 4,14 | 3,71 | 2,91 | 2,76 |
| 7 | Boxer | M | 4,83 | 2,91 | 0,95 | 5,08 | 7,07 | 6,03 | 5,01 | 3,79 |
| 7 | Chihuahua | F | 3,15 | 1,83 | 0,48 | 3,21 | 4,44 | 3,71 | 2,96 | 2,44 |
| S | Shar Pei | M | 3,67 | 2,06 | 1,61 | 3,73 | 5,44 | 5,33 | 3,68 | 3,00 |
| S | Shar Pei | M | 3,56 | 2,18 | 1,31 | 3,70 | 5,29 | 5,02 | 3,64 | 3,09 |
| S | Beagle | M | 3,72 | 2,06 | 1,34 | 3,49 | 5,31 | 4,82 | 3,64 | 2,95 |
| S | Beagle | M | 3,58 | 2,29 | 1,39 | 3,49 | 5,39 | 4,89 | 3,49 | 2,86 |
| S | Border Collie | F | 3,60 | 2,43 | 1,50 | 3,81 | 5,59 | 5,32 | 3,70 | 2,97 |
| S | Jack Russell T. | F | 3,09 | 1,98 | 1,44 | 2,98 | 4,68 | 4,41 | 3,02 | 2,62 |
| S | Jack Russell T. | M | 3,13 | 1,72 | 1,13 | 3,11 | 4,49 | 4,23 | 2,85 | 2,52 |
| S | Jack Russell T. | F | 2,68 | 1,80 | 0,66 | 2,76 | 3,93 | 3,39 | 2,41 | 2,44 |
| S | Jack Russell T. | M | 2,94 | 1,53 | 0,66 | 3,07 | 4,13 | 3,73 | 2,86 | 2,52 |
| S | Jack Russell T. | F | 3,22 | 2,13 | 1,27 | 3,31 | 4,90 | 4,57 | 3,05 | 2,71 |
| S | Pinscher | F | 2,61 | 1,39 | 0,85 | 2,58 | 3,64 | 3,43 | 2,33 | 2,27 |
| 0 | Shar Pei | F | 3,40 | 2,30 | 1,28 | 3,74 | 5,18 | 5,00 | 3,67 | 2,94 |
| 0 | American Cocker Sp. | M | 3,79 | 2,23 | 1,65 | 3,59 | 5,60 | 5,24 | 3,89 | 3,13 |
| 4 | Labrador R. | M | 3,64 | 2,13 | 1,33 | 3,38 | 5,22 | 4,71 | 3,58 | 2,69 |
| 4 | Labrador R. | M | 3,50 | 1,84 | 1,36 | 3,32 | 5,03 | 4,67 | 3,40 | 2,83 |
| 4 | Golden R. | F | 3,74 | 2,54 | 1,51 | 4,12 | 5,76 | 5,62 | 3,92 | 2,92 |
| 3 | Labrador R. | M | 3,58 | 2,30 | 1,42 | 3,72 | 5,42 | 5,12 | 3,75 | 2,83 |
| 3 | Labrador R. | M | 3,57 | 2,20 | 1,23 | 3,64 | 5,18 | 4,87 | 3,56 | 2,68 |
| 4 | Alaskan Malamute | M | 4,14 | 2,60 | 1,56 | 4,23 | 6,20 | 5,80 | 4,10 | 3,28 |
| 3 | Labrador R. | F | 3,74 | 2,53 | 1,46 | 3,79 | 5,72 | 5,25 | 3,80 | 3,06 |
| 3 | Labrador R. | M | 3,94 | 2,46 | 1,36 | 4,11 | 5,91 | 5,47 | 4,04 | 3,11 |
| 3 | Labrador R. | M | 3,77 | 2,42 | 1,30 | 3,95 | 5,65 | 5,25 | 3,85 | 13,10 |
| 3 | Leonberger | F | 4,17 | 2,69 | 1,61 | 4,34 | 6,32 | 5,95 | 4,14 | 3,36 |
| 3 | Leonberger | M | 4,02 | 2,74 | 1,33 | 4,17 | 6,13 | 5,60 | 4,01 | 3,11 |
| 4 | Leonberger | M | 3,85 | 2,66 | 1,42 | 4,18 | 5,96 | 5,57 | 3,95 | 3,24 |
| 4 | American Cocker Sp. | M | 3,38 | 2,03 | 1,25 | 3,41 | 4,93 | 4,66 | 3,36 | 2,81 |
| 4 | Pinscher | F | 2,75 | 1,43 | 0,72 | 2,81 | 3,86 | 3,50 | 2,62 | 2,36 |
| 5 | Leonberger | M | 4,93 | 2,87 | 1,28 | 5,08 | 7,37 | 6,35 | 4,70 | 3,80 |
| 7 | Leonberger | M | 3,90 | 1,93 | 1,53 | 3,63 | 5,13 | 5,16 | 3,91 | 3,05 |
| 7 | Leonberger | M | 4,05 | 2,39 | 1,65 | 3,84 | 5,84 | 5,48 | 3,88 | 3,07 |
| S | Eng. Setter | F | 3,70 | 2,26 | 1,45 | 3,87 | 5,64 | 5,30 | 3,60 | 2,95 |
| S | Springer Sp. | M | 3,59 | 2,02 | 1,24 | 3,55 | 5,18 | 4,79 | 3,37 | 2,89 |
| S | Springer Sp. | M | 3,39 | 2,12 | 1,28 | 3,52 | 5,00 | 4,80 | 3,27 | 2,87 |
| S | Springer Sp. | F | 3,44 | 2,21 | 1,17 | 3,34 | 5,10 | 4,53 | 3,27 | 2,86 |
| 0 | Dachshund | F | 2,53 | 1,75 | 0,77 | 3,08 | 4,02 | 3,85 | 2,80 | 2,24 |
| 0 | Whippet | F | 3,31 | 2,20 | 1,39 | 3,44 | 5,04 | 3,44 | 3,07 | 2,78 |
| 3 | Afghan Hound | F | 3,56 | 2,50 | 1,92 | 3,55 | 5,67 | 5,49 | 3,35 | 2,75 |
| 4 | Afghan Hound | M | 3,86 | 2,39 | 1,47 | 3,92 | 5,82 | 5,38 | 3,54 | 2,80 |
| 4 | Afghan Hound | M | 3,97 | 2,76 | 1,63 | 4,15 | 6,19 | 5,76 | 3,63 | 2,86 |
| 3 | Afghan Hound | F | 3,65 | 2,72 | 1,65 | 3,89 | 5,86 | 5,53 | 3,72 | 3,00 |
| 3 | Afghan Hound | F | 3,82 | 2,70 | 1,82 | 3,88 | 6,09 | 5,72 | 3,85 | 2,99 |
| 3 | German Shepherd | M | 4,02 | 2,63 | 1,94 | 4,01 | 6,24 | 5,96 | 4,14 | 3,09 |
| 4 | German Shepherd | F | 3,68 | 2,12 | 1,13 | 3,61 | 5,28 | 4,74 | 3,37 | 2,90 |
| 3 | German Shepherd | F | 3,65 | 2,68 | 1,50 | 3,99 | 5,89 | 5,49 | 3,55 | 2,83 |
| 4 | Hovavart | F | 3,71 | 2,46 | 1,85 | 3,98 | 5,81 | 5,82 | 3,61 | 2,96 |
| 3 | Schnauzer giant | F | 3,66 | 2,26 | 1,90 | 3,34 | 5,50 | 5,17 | 3,41 | 2,86 |
| 4 | Schnauzer giant | F | 3,64 | 2,34 | 1,54 | 3,53 | 5,55 | 5,07 | 3,47 | 2,97 |
| 3 | Schnauzer giant | F | 3,85 | 2,42 | 1,49 | 3,83 | 5,59 | 5,30 | 3,75 | 3,15 |
| 4 | Schnauzer giant | M | 3,80 | 2,48 | 1,35 | 3,80 | 5,73 | 5,16 | 3,61 | 3,04 |
| 3 | Schnauzer giant | F | 3,82 | 2,29 | 1,46 | 3,88 | 5,66 | 5,33 | 3,65 | 3,04 |
| 4 | Eng. Setter | F | 2,91 | 2,52 | 1,33 | 3,18 | 4,85 | 4,52 | 3,22 | 2,75 |
| 4 | Eng. Setter | F | 3,59 | 2,47 | 1,71 | 3,63 | 5,67 | 5,34 | 3,75 | 3,00 |
| 4 | Eng. Setter | M | 2,99 | 2,49 | 1,27 | 3,45 | 5,00 | 4,70 | 3,23 | 2,85 |
| 5 | Saint Bernard | F | 4,24 | 2,89 | 1,85 | 4,33 | 6,46 | 6,18 | 4,54 | 3,62 |
| S | Jagd T. | M | 3,20 | 2,37 | 1,48 | 3,33 | 5,17 | 4,77 | 3,38 | 2,69 |
| S | Jagd T. | M | 3,27 | 2,49 | 1,77 | 2,94 | 5,11 | 4,69 | 3,27 | 2,71 |
| S | Jagd T. | M | 3,19 | 2,24 | 1,55 | 3,22 | 5,05 | 4,72 | 3,25 | 2,68 |
| S | Jagd T. | M | 3,23 | 2,07 | 1,49 | 2,99 | 4,90 | 4,46 | 3,20 | 2,89 |
| S | Maremma Sheepdog | F | 3,56 | 1,73 | 1,29 | 3,48 | 5,05 | 4,77 | 3,59 | 2,95 |
| 3 | Maremma Sheepdog | M | 4,20 | 3,00 | 1,88 | 4,34 | 6,52 | 6,18 | 4,21 | 3,35 |
| 3 | Maremma Sheepdog | F | 4,28 | 2,56 | 1,66 | 4,22 | 6,17 | 5,86 | 4,08 | 3,16 |
| 4 | Maremma Sheepdog | M | 4,02 | 2,93 | 1,70 | 4,19 | 5,42 | 5,89 | 3,92 | 3,22 |
| 2 | Belgian Shepherd | M | 3,58 | 1,54 | 0,87 | 3,68 | 4,94 | 4,56 | 3,19 | 2,69 |
| 7 | Maremma Sheepdog | F | 3,66 | 2,58 | 1,55 | 3,84 | 5,72 | 5,39 | 3,73 | 3,07 |
| 7 | Maremma Sheepdog | F | 3,98 | 2,61 | 1,76 | 3,98 | 6,05 | 5,73 | 3,78 | 3,09 |
| 0 | Poodle toy | F | 2,90 | 1,91 | MD | MD | 4,12 | MD | MD | MD |
| 2 | Poodle toy | M | 2,98 | 1,76 | 0,99 | 2,91 | 4,30 | 3,90 | 2,70 | 2,45 |
| 2 | Poodle toy | M | 2,94 | 1,72 | 0,93 | 2,88 | 4,24 | 3,81 | 2,70 | 2,49 |
| 3 | Poodle toy | F | 2,91 | 1,49 | 1,08 | 2,80 | 4,08 | 3,88 | 2,60 | 2,46 |
| 3 | Poodle toy | F | 2,75 | 1,62 | 0,92 | 2,82 | 3,96 | 3,74 | 2,43 | 2,28 |
| 4 | Poodle toy | F | 2,95 | 1,88 | 1,15 | 2,93 | 4,45 | 4,09 | 2,88 | 2,54 |
| 5 | Bull T. mini | M | 3,39 | 2,26 | 1,26 | 3,35 | 5,09 | 4,61 | 3,27 | 2,73 |
| 8 | Poodle toy | F | 2,91 | 1,77 | 0,91 | 2,79 | 4,21 | 3,68 | 2,44 | 2,41 |
| 8 | Poodle toy | M | 2,87 | 1,62 | 0,92 | 2,82 | 5,05 | 4,77 | 3,59 | 2,95 |
